# Infrastructuring an organizational node for a federated research and data network: A case study from a sociotechnical perspective

**DOI:** 10.1017/cts.2021.846

**Published:** 2021-09-13

**Authors:** Marcelline R. Harris, Lisa A. Ferguson, Airong Luo

**Affiliations:** 1 School of Nursing, University of Michigan, Ann Arbor, MI, USA; 2 School of Medicine, University of Michigan, Ann Arbor, MI, USA; 3 Consultant, EduLand LLC, Wexford, PA, USA

**Keywords:** Infrastructuring, federated research networks, PCORnet, sociotechnical, data networks, network node

## Abstract

**Background:**

Local nodes on federated research and data networks (FR&DNs) provide enabling infrastructure for collaborative clinical and translational research. Studies in other fields note that infrastructuring, that is, work to identify and negotiate relationships among people, technologies, and organizations, is invisible, unplanned, and undervalued. This may explain the limited literature on nodes in FR&DNs in health care.

**Methods:**

A retrospective case study of one PCORnet® node explored 3 questions: (1) how were components of infrastructure assembled; (2) what specific work was required; and (3) what theoretically grounded, pragmatic questions should be considered when infrastructuring a node for sustainability. Artifacts, work efforts, and interviews generated during node development and implementation were reviewed. A sociotechnical lens was applied to the analysis. Validity was established with internal and external partners.

**Results:**

Resources, services, and expertise needed to establish the node existed within the organization, but were scattered across work units. Aligning, mediating, and institutionalizing for sustainability among network and organizational teams, governance, and priorities consumed more work efforts than deploying technical aspects of the node. A theoretically based set of questions relevant to infrastructuring a node was developed and organized within a framework of infrastructuring emphasizing enacting technology, organizing work, and institutionalizing; validity was established with internal and external partners.

**Conclusions:**

FR&DNs are expanding; we provide a sociotechnical perspective on infrastructuring a node. Future research should evaluate the applicability of the framework and questions to other node and network configurations, and more broadly the infrastructuring required to enable and support federated clinical and translational science.

## Introduction

### Federated Research and Data Networks

For over a decade, the scientific environment has been characterized as data-intensive, dynamic, and fast-paced; this offers many opportunities and at the same time presents significant challenges [1]. Federated research networks have emerged as one model for advancing research in this new environment. While definitions of federated research networks vary, there is general consensus that federated research networks are essentially collaborations among partners who, through coordination at an overarching network level, bring together, share, and optimize resources and services in order to enable research that exploits this new data-intensive and connected scientific environment.

The term “federated research network” is sometimes used synonymously with “federated data network,” although federated data networks can be developed (in health care and other fields) for non-research purposes. In addition, some federated research networks rely on centralized data repositories rather than federated data networks. In this paper, we use the term “federated research and data network” (FR&DN) to indicate our focus on networks that incorporate the features of both a federated research network and a federated data network. FR&DNs are somewhat more established in other scientific disciplines (e.g., physical sciences, environmental sciences). A study of FR&DNs across scientific domains within the European Union noted that the network infrastructures must facilitate access, authentication, authorization, identification, linkage, pooling, sharing, interoperability, security, standards, legal/ethical issues, openness/publication, storage, and more broadly research data management in a distributed manner, but where data often reside at the local site [2]. A “major challenge” noted in that study of federated network infrastructures was the complex and somewhat fragmented nature of the environments in which the data and the research evolved.

Focusing on healthcare data networks, Weber speculated that given the widespread adoption of electronic health records (EHRs), it is likely that all of the 5500+ US hospitals will soon be connected to a federated data network [3]. The term federated data network implies that data are maintained in repositories at the local partner sites and that local data in those repositories are mapped to a common data model. Such an approach to modeling data for storage in a repository enables a broad range of queries to be pushed out centrally from the network and executed at the local level, without making changes to the query. Organizations hosting repositories on such a network also use a common technical network for transmitting queries and results. Emphasizing “federalist principles” for healthcare data networks, Mandl and Kohane called out the challenges encountered in instrumenting such systems locally, including the integration of top-down network approaches into the local system; harmonization of organization-specific data to a common data model and terminology; and the pace at which local IT departments can scale efforts within constrained budgets and competing priorities [4].

The National Patient-Centered Clinical Research Network (PCORnet®) is one example of a national scale FR&DN in health care (https://pcornet.org). Launched in 2013 with funding from the Patient-Centered Outcomes Research Institute (PCORI), PCORnet® originally described itself as a distributed research network [5]. By 2015, PCORnet® included 13 clinical data research networks (CDRNs), 20 patient-powered research networks (PPRNs), and two collaborating partners in the PCORnet® coordinating center – the Duke Clinical Research Institute and the Harvard Pilgrim Health Care Institute. The technical platform for PCORnet®, PopMedNet™, is an open-source technology specifically designed to support research data networks [6]. Each CDRN also included a number of participating organizations. In this paper, we use the term “node” to refer to these distal organizations within PCORnet® that host data repositories, are partners in a CDRN, and participate in network-based research. At the completion of Phases I and II of establishing PCORnet®, there were 13 CDRNs that included over 80 nodes, each node tasked with creating, maintaining, and standardizing data to support PCORnet® research, and engaging local stakeholders in the research.

At the time this paper was written, PCORnet® described itself as a network of research networks and included nine clinical research networks (CRNs), two health plan research networks (HPRNs), and the same two partner organizations in a coordinating center (https://pcornet.org/about/). The change from "CDRN" to "CRN" was perhaps motivated by an effort to emphasize the research network and not only the data network. We use the abbreviation "CDRN" throughout this paper to reflect PCORnet®’s term in use at the time of this study. Throughout the evolution of PCORnet®, the assumption has been that individual nodes within CDRNs would leverage internal resources and services to meet the requirements and milestones put forward by the network, although the scope of resources, services, and projected expenses was never explicitly stated.

While PCORnet was one of the early FR&DNs in health care, a growing body of literature describes other federated research networks in health care that also leverage federated data networks. Across this literature, there is an emphasis on *network-level* technical infrastructures, governance and regulatory components, collaboration, and benefits to the science [7–13]. An entire issue of the Journal of the American Medical Informatics Association (July 2014) was dedicated to describing the development of CDRNs within PCORnet® [14–24]. Across these publications, the heterogeneity of collaborating organizations is noted as a strength of the network, and the general methods and technologies used to implement queries across the network are described. However, these publications have a limited focus on the extensive work required by local organizations to engage in such networks.

**Fig. 1.**
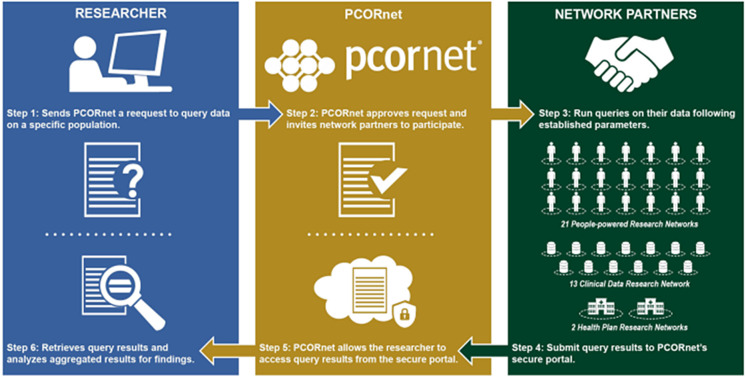
PCORnet® research data query-response workflows across the network: an example of not recognizing nodes participating in federated research and data networks. *Source:* GAO analysis of Patient-Centered Outcomes Research Institute information. GAO-18-311.

As another example, Fig. 1 below was published in a Government Accounting Office report and illustrates the processes by which a research query submitted by a researcher makes its way through the PCORnet® network https://www.gao.gov/assets/gao-18-311.pdf [25]. Note that while CDRNs are represented in the graphic, there is no depiction of the individual nodes that are a component of each CDRN; the nodes on the network are not visible.

Organizational-level nodes serve as the interface among all queries of the data repositories (a.k.a data marts), research studies, researchers, and multiple levels of governance. Those establishing a node must not only assemble local resources, services, and expertise, but also navigate the complex configurations, relationships, and governance across multiple organizational entities including the following: the local organization, the data networks, and research networks at regional levels such as CDRNs, at national levels such as PCORnet®, and across the multiple research teams conducting studies. The complexity facing individual organizations hosting a node within a larger FR&DN can expand greatly when organizations participate in multiple federated research and data networks. Participation in each network requires an interconnectedness of work efforts across potentially different scales, focus, governance and regulations, technical platforms, approaches to data use and sharing, funding models, and more.

**Fig. 2.**
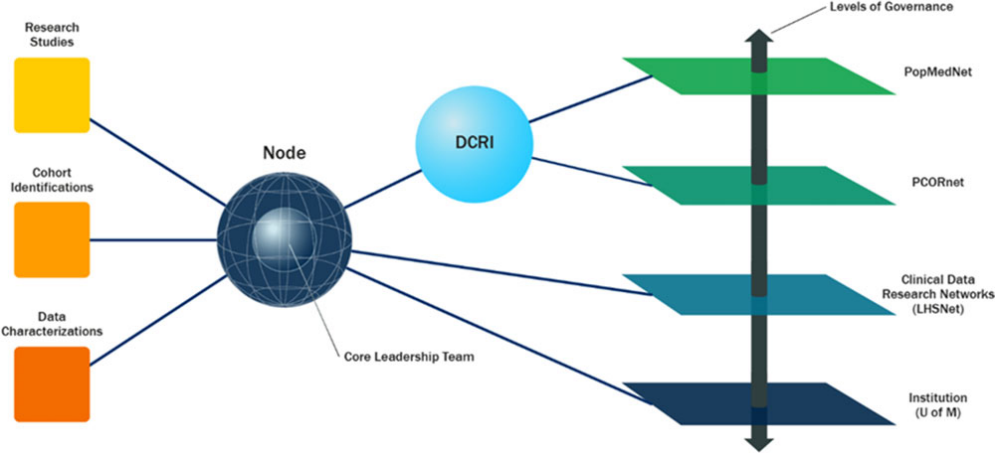
Centrality of the node in studies, data queries, and multiple levels of governance.

Further adding to the challenges faced at the local level is the heterogeneous nature of the organizations that host nodes, sometimes making it difficult to share approaches and solutions among these diverse organizations. As an example, while the coordinating centers within PCORnet® facilitate forums in which nodes can communicate with each other on various topics, the uniqueness of each organization often precludes full adoption of successful strategies and approaches that were used by a specific organization. Proprietary data models associated with specific EHR vendors, local implementation of documentation systems and data repositories, local policies related to compliance, regulatory guidelines, technical security models, and local structures and practices supporting research all represent limitations to the reuse of specific implementation strategies across nodes. Furthermore, nodes vary widely with respect to the availability of people and expertise required to accomplish key tasks, data storage capacity, and software availability and expertise (e.g., required statistical packages). Figure 2, below, illustrates the centrality of the local node in executing the on-the-ground work required to achieve the goals of the PCORnet® network.

### A Sociotechnical Perspective of Infrastructure and Infrastructuring

The literature on FR&DNs in health care is limited with respect to the development of node infrastructures, but for over 20 years the nature of the infrastructure that enables collaborative, networked, and large-scale science (e.g., cyberinfrastructure) has been examined from a sociotechnical perspective. Cyberinfrastructures bring together people, information, technologies, data repositories, and computational services to support research [26,27]. In a now classic article, Star and Ruhleder argued that it is neither useful nor accurate to characterize such infrastructure as static systems that are built and maintained to serve as "scaffolding" on which things operate [28]. Rather, from a sociotechnical perspective, they argued that infrastructure is fundamentally a *relational* concept. Infrastructure is characterized by its embeddedness in social, technical, and organizational structures, transparency in supporting tasks, having both spatial and temporal reach or scope, learned as a part of membership within a community, shaped by conventions of practice, plugged into other infrastructure through the embodiment of standards, operating on an installed base, and visible upon breakdown [28]. Infrastructures are further described as sociotechnical assemblies of objects that support the *doing* of science and include the entirety of devices, tools, technologies, standards, conventions, protocols, policies, and the relationships needed among people, organizations, and technologies that are relied on to carry out tasks and achieve goals [27,29].

The dynamic, open-ended, and active processes of creating, managing, and facilitating the relationships needed to create infrastructure arereferred to as “*infrastructuring*.” Infrastructuring involves sociotechnical arrangements wherein ‘technical, political, legal, and/or social innovations link previously separate, heterogeneous systems to form more powerful and far-reaching networks” [30]. Researchers have further identified and highlighted concerns that both infrastructure and infrastructuring typically exist in the background, are invisible, and are frequently taken for granted. Both the work and the workers involved in infrastructuring are subsequently easily undervalued, underfunded, and marginalized [31,27].

Ribes and Finholt proposed a framework that addresses dimensions of infrastructuring across three dimensions: enabling technology, organizing work, and institutionalizing [32,33]. They further identified tensions that often manifest when considering the sustainability of the infrastructure across these dimensions, particularly around aligning end goals, motivating and ensuring participation, and designing for use and adoption. Noticeably, many of the tensions that Ribes and Finholt identified in studies of cyberinfrastructures were motivated by observations that are strikingly similar to the concerns expressed about the challenges experienced when instrumenting for federated research and data networks in health care [3,4]. Like other researchers studying infrastructuring, Ribes and Finholt argue that analyzing infrastructuring requires looking at the full breadth of activities involved in infrastructuring.

### Infrastructuring a Node on PCORnet®: A Case Study

The purpose of this retrospective case study was to examine, from a sociotechnical perspective, infrastructuring for organizational participation as a node on a PCORnet® CDRN. Our research questions, directed at the organizational node level, were (1) how were infrastructure components (e.g., expertise, data, technologies, procedures) assembled and coordinated to meet the network milestones; (2) what were the specific work efforts for infrastructuring that were undertaken by the teams to meet network, CDRN, and organizational requirements; and (3) informed by question 1 and 2, could we identify generalizable sociotechnical considerations that should be considered before undertaking the infrastructuring of a node on a federated research network. Our goals were to discern the work required at the organization/node level to ensure that all milestones were achieved at the CDRN level and to provide a framework and guide for applying a sociotechnical perspective of infrastructuring, which is absent in the literature of FR&DNs in health care.

## Materials and Methods

### Case Study

We applied a case study methodology as described by Yin (2009) [34]. A retrospective, single-case analysis of the infrastructuring was conducted, with a focus on the University of Michigan’s experience of establishing a node on PCORnet® and the LHSNet CDRN. A description of LHSNet, a CDRN on PCORnet®, was published previously but similar to other reports about CDRNs, did not report on the work of the individual nodes within the CDRN [35]. The specific PCORI-funded initiative that is examined here spanned a 3-year timeframe supported in part by PCORnet’s® Phase II CDRN awards (2015–2018).

### Procedures

Data were obtained from various sources, including direct observations and notes by participant-observers; semi-structured interviews with internal stakeholders and external partners; documents stored at the local, CDRN, and network level (e.g., minutes, emails, and other types of documents). Two authors (MRH and LAF) were participant-observers in this study. As members of the core leadership team, they had key responsibilities for the design and implementation of the U-M node. The third author (AL) is a social scientist who was embedded in the organizational technology team that provided technical support for research overall and the node. One or more authors participated in all project activities such as identifying and procuring local resources; leading, coordinating, and convening working groups; developing and/or customizing policies and procedures; and ensuring appropriate documentation. In addition, one or more authors participated in all meetings spanning the multiple organizations and organizational levels of the project including with LHSNet partners, PCORnet®, and PopMedNet™. Colleagues within the U-M organizational environment, as well as colleagues from other nodes within the same CDRN (LHSNet), were engaged to review data and confirm that our interpretations were consistent with their perceptions of the work in which they engaged.

We reviewed documentation and artifacts from the early period of node implementation planning and launch, specifically focusing on the local groups that were formed to meet the initial milestones published by PCORnet®, and the collaboration that was required to meet each milestone. Note that while the PCORnet® milestones targeted the CDRN level of the federation, evaluations of the CDRN’s readiness to be “approved for research” included parameters that had be met at the node level – technical parameters, research participation parameters, and stakeholder engagement parameters. But at CDRN levels, there was no direct visibility into the distal nodes that hosted the data repositories recruited stakeholders and implemented the studies locally.

After review of the documents and artifacts, we used standard mind-mapping approaches to categorize the activities and graphically represent the resulting classification of efforts. Organizational faculty and staff who were directly involved in the design and building of the node reviewed the classification of work efforts to validate and/or suggest additional detail that we may not have included. We also asked those individuals to clarify and confirm which components of the infrastructure previously existed and had been leveraged for the node work (i.e., the "installed base" such as technical resources, existing artifacts, processes, expertise) and to surface any new resources or work that was required to establish the node.

Applying the Ribes and Finholt framework of infrastructuring [32,33], we next sought to identify theoretically grounded, pragmatic questions that organizations might consider prior to engaging in node development. As validation, we reviewed all of the questions with colleagues involved in establishing other nodes on the same CDRN, asking “Do these questions reflect what you had to think about as you established a node?” and, “Have we missed anything?”

## Results

The work of infrastructuring at the node level required alignment with organizational strategies as well as collaboration and coordination among members of new teams that were created to address the immediate, time-sensitive work required to meet specific milestones. A core leadership team was comprised of the principal and co-investigators, and a project manager with deep experience in EHRs and an advanced degree in interdisciplinary information science. The leadership team met weekly and assumed the operational aspects of the project including the identification, engagement, and as necessary, recruitment of required expertise (e.g., requesting effort from existing teams, hiring new people, or engaging consultants). A steering committee comprised of high-level leadership from across the organization was convened and met monthly to consider and advise on strategic decisions that were required to align organizational policies and procedures with network policies and procedures. A technology and informatics team met weekly, although additional meetings were typically needed throughout the implementation. This team included a data architect, two research informaticists, a statistical programmer analyst, and database developers and administrators. A research study team and an engagement team included researchers and patient-level stakeholders, and focused on the extent to which the milestones were aligned with existing research practices and patient engagement. Each team was given maximum flexibility to develop strategies and procedures that would enable them to meet the milestones within the required timeframes. The project manager participated in every team. Members of the core leadership team frequently met with representatives of organizational departments that supported research to work through alignment of existing policies with network requirements (e.g., streamlined IRB language and reliance agreements, technical security considerations when connecting the local node’s data mart to the Distributed Research Network Architecture of PopMedNet™). Table 1 reflects the collaboration that was required across node-level teams to ensure that our node met the required milestones to be designated as approved for research.

**Table 1.** PCORnet® CDRN milestones and local team collaborations

		Local teams contributing to CDRN milestones				
CDRN milestone & descriptions		Leadership team	Steering committee	Technology & informatics team	Study teams	Engagement team
1	Governance: Materials that describe and support network governance (e.g., charters, policies)	X	X			X
2	Institutional Review Board (IRB) Approval: All network sites have obtained IRB review/approval for the overall application	X				
3	Engagement Strategies: Patient and Engagement Stakeholder Plans submitted	X	X		X	X
4	PCORnet Common Data Model (CDM): Map data to PCORnet Common Data Model (CDM)	X		X	x	
5	PCORnet Distributed Research Network (DRN) Architecture: Instantiate PopMedNet™ as tool for executing queries	X		X		
6	Basic PCORnet Query Capability: Demonstrate ability to execute a query against real data	X		X		
7	Computable Phenotypes: Create and validate algorithms to identify condition-specific cohorts	X		X	X	X
8	Cohort Creation: establish 3 cohorts (common, rare, weight)	X		X	X	X
9	Data Quality: execute data quality/data characterization programs	X		X	X	X
10	PCORnet Collaboration: Partnerships with other CDRNs or PPRNs	X			X	X
11	Research Integration: Demonstrate ability to use health system resources to support research	X	X		X	X
12	Streamlined IRB capability: Master Reliance Agreement in place	X				
13	Rapid Start-up Capability: Deploy approaches to streamline research operations and processes	X			X	X
14	Informatics Innovation: Test novel informatics capabilities to support network research	X		X		
15	Data Linkages: Demonstrate progress in claims linkage data to EHR data	X	X	X		
16	Study Participation: Participate in formally designed PCORnet study	X	X	X	X	X
17	PCORnet CDM Enhancement: Contribute new data elements to the CDM	X		X		
18	Data Completeness: Complete linkage of claims data to clinical data	X		X		
19	Data Governance: Data governance approach in place (e.g., data sharing policies, data use agreements)	X		X		
20	Rapid Response Capability: Develop capability to respond efficiently to approximately 200 queries annually executed against quality-checked datasets	X		X		
21	Collaborative Community: Demonstrate willingness to participate in multiple PCORnet studies, including those led by researchers from outside CDRN	X			X	X
22	Sustainability: Submit a sustainability plan	X	X			
23	Collaborative Community: Participate in PCORnet-wide activities and task forces		X		X	X
24	Collaborative Community: Disseminate open-source software, computable phenotype algorithms, and other technologies within PCORnet and broadly	X		X		

Our analysis of the discrete efforts that were included in the final classification of work efforts revealed that of the 148 discrete work efforts we identified, fewer than half (43%) directly enabled the technical infrastructure. Note this count reflects tasks; it does not equate or suggest a count of personnel or hours needed to accomplish those tasks (See Table 2 for examples). The majority of the work efforts involved the relational work of infrastructuring. This included work such as aligning and mediating the governance of the node across the organization, the CDRN, and PCORnet®, motivating contributions to the node in the context of competing priorities for local teams, coordinating expertise and resources across organizational units, developing and aligning policies and practices within and across organizational units, and providing just-in-time support to site-level researchers on the operational features of a federated research network.

**Table 2.** Local teams, charge, membership and responsibilities

Team, charge, & membership	Key responsibilities
**Core Leadership Team** Charge: Managing body to drive project to completion and beyond (e.g., turnover to ongoing operations) Membership: Site Principal Investigator (PI) Informatics Co-Investigator (Co-I) Research Co-I Engagement Co-I Project Manager	Manage all facets of the project including Governance, Management, Technology, Regulatory, Research, and Patient/Stakeholder Engagement Function as the communication arm to the local project Steering Committee Focal point for local project decision-making and escalation of project issues Ensure effective teamwork across the project Coordinate and assemble required resources Ensure appropriate stakeholders are at the table in the appropriate forums Oversee execution to the project schedule Prevent “interrupts” from other projects/initiatives Responsible for implementing workarounds if interrupts cannot be avoided Identify risks and execute risk mitigation plan(s) as needed Act as fast decision-making mechanism to accelerate the project Approve items to be brought to the project Steering Committee Develop and document operational processes, procedures, and workflow Act as local node liaison with the CDRN leadership and workgroups Provide required reporting internally, as well as externally to network partners and others
**Steering Committee** Charge: Overall governance, Advise, and endorse decisions regarding project scope, schedules, and resources, Help resolve escalated issues and risks Membership: Chief Information Officer Chief Medical Information Officer Institutional Data Warehouse/Analytics Director Electronic Medical Record System/Research Liaison Health Policy Institute Health Services Researcher Director, Office of Research Director, Michigan Institute for Data Science Director, Michigan Institute for Clinical and Health Research (CTSA) Patient Advisor (Recruited by Office of Patient Experience) Core Leadership Team	Bring viewpoints of all key stakeholders to bear on decisions Provide oversight/reviewing progress toward meeting goals and deliverables Make decisions on items pertaining to the entirety of the project and its plans Make decisions about specific items brought forward by Core Leadership Team and other Teams Assist in identifying and engaging resources needed for the project Serve as a sounding board for issues and problems Develop a strategy for long-term sustainability
**Technology and Informatics Team** Charge: Implement, monitor, and maintain the node’s technical system to meet requirements of PCORnet® data mart Membership:	(See below for responsibilities associated with each role) – tightly coupled work efforts, but individual work (These are the people doing the “invisible work” of infrastructuring for a FR&DN)
Informatics Co-Investigator	Lead the activities of the Technology and Informatics team toward developing infrastructure, processes, and policies to meet network milestones
Project Manager	Plan and oversee activities, tasks, and deliverables of Technology and Informatics team to ensure they are completed on time Organize project resources, prepare budgets, monitor progress, and keep stakeholders informed
DBA/Systems Architect	Design system Create & populate new tables as required by evolving data models and refreshes Define ETL processes to load data from source system(s) to relational and SAS DataMart Update and maintain SAS DataMart
Analyst	Respond to Front Door queries, maintain documentation Validate the queries against the target data model, the goals of the research, and any business rules that may constrain the return of the results Communicate with other data partners on query execution Execute SAS queries Execute SQL queries Create an analytic data set when sources outside of the target data mart are required
Clinical Research Informaticist (Senior Level)	Oversee data processes locally and collaboratively with the network Direct data integration locally Develop tools Contribute to data quality metric development at both the local and network levels Provide input to targeted network data model expansion and enhancements Support and understand requirements for scientific processes (research life cycle) Contribute informatics considerations to strategy and tactics that enable successful federated research (e.g., architecture, data linkage technical capabilities, data quality, and data standards)
Research Business Analyst	Prepare local documentation of relevant parameters needed to assess data quality for research use Monitor and report on local Data Quality Use existing tools and resources to support findability and accessibility of data quality for users of the local data mart Analyze and propose local research workflows based on the requirements of the networked research
Statistical Analyst	Provide support on study design, database, and statistical methodology Responsible for programming using SAS and providing statistical analysis
Domain Experts & Practitioners	Is the definitive source of knowledge or expertise in a specific area and applies this expertise to support the organization Advises on local domain-specific knowledge (e.g., Pharmacy, Health Information Management, Michigan Data Collaborative, Clinicians) and the business rules and other processes across the data stream from data capture through secondary use within the network
Data Manager	Ensures data collected is accurate, groups data properly, solves operational problems, and prepares statistical reports
Data Source Team Lead(s)	Responsible for helping to inform proper data sourcing for DataMart (e.g., mapping) Help with troubleshooting data quality and other data-related issues found in the DataMart
**Research Teams – supporting each study** Charge: Work together in a committed way toward a common research goal using the network and support Membership: Node Research Co-I (liaison from Core Leadership Team) Node Project Manager from Core Leadership Team Technology & Informatics collaborators as needed Study Specific Research Teams	Node Research Co-I: A member of the Core Leadership Team, acted as a liaison from the Core Leadership Team to the institutional research community Node Project Manager: A member of the Core Leadership Team, acted as a liaison to the specific study teams to help navigate the requirements and nuances of study participation using the network, as well as reusable artifacts (e.g., IRB and data sharing, data use language and agreements) Study Specific Research Team: Bound by the parameters of a specific study, composed of the same roles and responsibilities that would apply to any type of research study Technology and Informatics: advised on boundaries around CDM, and the technical aspects of the network and access
**Patient and Stakeholder Engagement Team** Charge: Engage patients and other key stakeholders to inform and advise the local node regarding participation in network-based research. Membership: Site Principal Investigator (PI) Informatics Co-Investigator (Co-I) Research Co-I Engagement Co-I Project Manager Patient Advisors (PA) PA for local Technology Informatics Team PA (2) for local Research Team PA for local Steering Committee PA for ADAPTABLE study (LHSNet Adaptor)	Engage those in the local and LHSNet community, specifically patients, clinicians, payers, and health system leaders regarding considerations specific to network-based research. Provide and/or solicit insights about healthcare issues, barriers, and topics that are of concern and interest to patients, clinicians, payers, and other stakeholders. Gather feedback on the research process, advise and facilitate dissemination of research results. Work cooperatively with the LHSNet Engagement Strategies and other CDRN workgroups and partner nodes to create and sustain infrastructure that can inform all phases of the research cycle. Inform and develop both local and CDRN engagement policies and processes (e.g., Patient Selection and Compensation Guidelines)

Resolving inevitable differences most often was accomplished through discussion, with an eye toward solutions that could be generally applied across future studies and collaborators. Changes to "standard operating procedures" were documented, and the core leadership team (especially the project manager) provided just-in-time advice for newly forming research teams. Among the most complicated work was aligning data use agreements, mapping local data to the common data model, and trying to fashion-together existing practices, policies, resources, and expertise that worked well in support of traditional multi-site research efforts, but now had to be reconfigured for the FR&DN research effort.

As a specific example, Milestone # 19 for the CDRN required that data governance approaches were in place (e.g., data sharing policies, data use agreements) and assumed that state and local regulations around data sharing were similar among nodes in the CDRN. We (and other nodes in our multi-state CDRN network) encountered state-level regulations that did not permit individual nodes to fully comply with the initial data sharing agreement provided by PCORnet®. Extensive discussions among legal and compliance representatives from the nodes, the CDRN, and PCORnet® were required to arrive at language that accommodated regulations that applied to individual nodes. As another example, Milestone #4 required mapping local data to the common data elements of the PCORnet® Common Data Model (CDM). This was a particularly complex milestone to meet, data harmonization in the absence of consistent documentation of local data provenance, and prior mapping to standard terminologies, combined with sometimes vague or absent definitions within the CDM (e.g., "patient" or "client" is never defined), created challenges that were difficult to overcome. As mentioned previously, across our CDRN, electronic health record (EHR) vendors raised proprietary concerns related to sharing information such as data models that underlie the EHR and reporting structures at a specific organization. In addition, there were notable differences in vocabulary and master data management practices at each organization that made it difficult to share approaches to mapping local data to the CDM. (We note here that initiatives such as Health Level 7’s Fast Healthcare Interoperability Resources (FHIR) https://www.hl7.org/fhir/, and the National Center for Data to Health (CD2H) https://cd2h.org/data_sharing address such data and terminology challenges through unifying standards and tools that enable terminology resources such as CDMs (including the PCORnet® CDM) to be integrated and extended locally; these are likely to be of high value at the node level).

At the time this node was launched, participation in federated research was new to the organization; socializing the paradigm of a FR&DN required extensive work. This included awareness activities, as well as persistent and repeated "just-in-time" discussions to reinforce the goals of the network, why the organization chose to participate in the network and what opportunities this participation offered for the organization, its researchers, and other involved stakeholders. Significant training and education were also required to ensure compliance with new policies and processes both locally and at the network level. Specific topics were important to all units within the organization including the underlying technology (e.g., the architecture of the local system and the network overall, the structure, and use of a common data model), regulatory-related information (e.g., the use of SMART IRBs, standard data use agreements), research practices (e.g., the development of new types of research questions and associated research budgets and resources), data management (e.g., data security, data retention, data use, data quality, data privacy, data linkages such as claims data to EHR data), and patient engagement (e.g., identifying important research questions, developing processes for research dissemination). Clarifying differences between federated research and data networks and multi-site research collaborations was critical to engaging stakeholders for infrastructuring efforts and to engaging local research teams in federated research studies.

Anticipating the organization’s participation in other federated data and/or federated research networks, decisions required constant consideration of the sustainability and reuse of the new assemblages of teams, the data mart, the mapping of local data to the network common data model, new template-based language for IRBs, compliance, and security reviews, and new types of stakeholder engagements. Table 2, below, provides details of the composition of the various U-M teams, their charge, and responsibilities at the local node level.

Jointly, Tables 1 and 2 provide representations of the “who, what, and why” of node infrastructure, but do not get to the “how” of infrastructuring. Questions addressing the “how” of infrastructuring emerged as we analyzed Tables 1 and 2 through a sociotechnical lens.

Table 3 provides a set of questions at the intersections of the dimensions of infrastructuring (columns) and sustainability considerations (rows). The framework and the questions reflect an adaptation of the theoretical literature on infrastructuring, applied to the actual experience of infrastructuring a node on a federated research network. Table 3 includes “real-world questions” that can lead to insights on the infrastructuring that is needed to plan, establish, and sustain a node for federated research networks.

**Table 3.** Sociotechnical considerations when infrastructuring for a federated research network node

Considerations	Sociotechnical dimensions of infrastructuring		
	*Enacting Technology* The practical activity of developing stable, usable infrastructure ([32])	*Organizing Work* The practical organizational arrangements, techniques, and routines for preserving and maintaining persistent infrastructure ([32])	*Institutionalizing* The practical work of generating sustainable infrastructure linked to social or collective purposes, with connotations of permanence, enduring beyond any particular scientific question ([32])
**Aligning End Goals: Sustain multiple ongoing goals that often compete**	How does constructing the technical aspects of a node align with existing organizational priorities? How will the local teams access and review the functional requirements and technical specifications developed by the network, and on which the node function is dependent? (e.g., software licensing fees such as SAS, high volume data storage needs, time to compute queries on large volume data) How will you engage and align existing expertise, and also local data collation, data standards, and data governance processes to accommodate network technical requirements? (e.g., assembling and sourcing data to populate the data mart, conforming to the common data model, mapping proprietary data models, security, compliance, data storage). How will you address the likely need to share sensitive information on platforms not provided by the Federated Research Network, but hosted by individual nodes? (e.g., data and information sharing between nodes, but not network-wide) If the internal technical and informatics capacity is limited (e.g., because of competing priorities), are there resources available to engage contractors, consultants, etc.?	Are the key stakeholders within the organization responsible and accountable for executing the type of work needed to partner with external entities such as Federated Research Networks? How will the node team identify, access, and engage organizational resources, processes, and expertise on which the node work is dependent? How will the organization identify and address potential conflicts between existing local policies and practices and network requirements? (e.g., data use agreements, data sharing, how the technical system works). How does this project synergize with other ongoing/planned initiatives so that it contributes to other organizational goals that require similar technical, informatics, and research expertise?	How does the organization embrace and commit to embedding and integrating infrastructural components developed from individual projects into the organizational infrastructure? (e.g., will the node work be embedded into research infrastructure that supports cross-network collaboration, or other types of collaborations?) How does the organization enable awareness and reuse of infrastructure and *infrastructuring processes* developed by individual projects? (e.g., enables the node to discover stuff that’s useful and others to discover node work that may be useful to them) What organizational processes are in place to inform projects of upstream changes that could affect the ongoing maintenance and sustainability of the downstream production system (e.g., system changes affecting secondary use of data (e.g., pharmacy, laboratory, and other clinical information systems)? When there are emergent or urgent competing needs (e.g., Pandemic response, major reimbursement and payment system changes), how will you sustain dedicated resources for the node?
**Motivating and ensuring participation: Ensuring participants contribute in ways that are meaningful to the achievement of community infrastructure**	How will the project team motivate contribution of internal resources and expertise, beyond what is provided through the start-up budget/funding? (e.g., demonstrating benefits to the organization, ) How will the project team leverage existing relationships with internal technical and informatics groups, or engage groups who could contribute needed expertise? (e.g., liaisons among IT departments and relevant clinical and business processes)	To ensure achieving both local and network goals, how will the organization facilitate communication and collaboration among internal work groups, stakeholders, and external communities? How will you engage decision-makers who need to approve actions and strategies that may alter or expand local processes in order to align with network requirements? (e.g., create new teams) How will your organization facilitate education and/or training on how a research network functions, including the benefits to researchers, the organization, and the network?	How will you sustain support for maturation of the research network infrastructure after the start-up period? How does your organization incentivize contribution to shared infrastructure and collaborative infrastructuring? What are the additional benefits for the organization, that is, beyond the internal benefits of participating as a node on a single research network? How will your organization develop the collective mindset needed to contribute and support resources and expertise that are broadly accessible, sustainable, and reusable and span single grant/single study to federated networks?
**Designing for Use:** **Develop resources that will be adopted by users and serve in the future work of research**	How might the data mart meet needs for structured and coded data beyond one Federated Research Network? How will you identify work products that can be modularized and reassembled as needed? (e.g., terminology management, common data elements, forms, processes) What level of information about the network infrastructure is available for review by the organization to understand what needs to be built locally? (e.g., can you anticipate impacts to local infrastructure such as data storage requirements?) What processes and documentation are required to support ongoing operations and enhancements of the local system? (e.g., query alignment and approval, data retention, compliance, information assurance, security, privacy)	How will the node identify and work with key groups in the organization to develop shareable artifacts such as IRB templates and data use agreements for federated research networks? What data stewardship processes will you use to review query results of the data mart (e.g., cohort identification, research studies) that require the engagement of various stakeholders (e.g., researchers, legal, compliance, IRB? What existing resources and expertise need to be assembled for the local technical and informatics components required to participate in the research network, and how will you assemble them? Considering data quality as “fit to purpose,” who should be involved in adjudicating any data quality concerns and/or performing any data audits? (e.g., verification and validation of all data queries) How will the organization engage patients, caregivers and other key stakeholders to provide input into federated research questions, methods, goals, objectives and other considerations (e.g., data privacy)?	How will people find and leverage the opportunities and resources that arise from node participation? How will you prepare people to participate in network level studies and ensure that the applicable policies and procedures are followed? In particular, how will you train people to understand the "federation" aspects (e.g., data are not centrally collated, use of common data model, new network policies and procedures). How can work processes from one project be scaled up for use in other networks? What documentation is available for reference by others? (e.g., for organizational memory, learning and supporting development of best practices, to avoid reinventing the wheel) What data governance strategies exist that encompass information architectures, semantics, data quality, data management and analytics *–* and is it sufficient for this purpose and reusable for others? What mechanisms, organizational structures or processes exist, or, can be instantiated, to facilitate patient engagement across multiple federated research networks?

The dimensions of infrastructuring include enacting technology (developing stable and usable infrastructure), organizing work (arrangements, routines, techniques), and institutionalizing (related to social or collective purposes and permanence). The sustainability considerations included aligning end goals among multiple goals that may compete, motivating and ensuring participation, and developing resources and expertise (human, organizational, and technical) that serve future uses. To assure some level of validation, we iterated the wording of questions with colleagues deeply involved in building our local node, as well as with colleagues at other nodes within the same CDRN, until we arrived at a stable set of questions and obtained agreement on the completeness of the questions for the work that was undertaken, and their placement within the framework.

## Discussion

Network-based research offers opportunities to accelerate and enhance research capacity, share technology and data platforms, and facilitate collaborations among multiple stakeholders. In this case study, we analyzed the work of infrastructuring one local node on one network, and from that analysis generated a set of questions for others to consider when preparing for infrastructuring a node on a federated research network. Existing studies have focused on the networks themselves; we contribute a focus on the work of infrastructuring a local node for participation in the network.

At a granular level, we found that time-consuming mediation by individuals on the node leadership team and steering committee was required at many different levels of governance, and between many different individuals and groups including between the local node and the network; between different disciplines; and between regulations and policies that varied across states, the local organization, and work units within the organization. Existing but somewhat siloed resources, services, and expertise needed to be engaged, assembled, and embedded into the specific workflows required to meet the requirements that enabled CDRN participation in the network. Researchers, studies, and teams across the organization and levels of the network had dependencies on people within local work units that were simultaneously contributing to other, and sometimes competing, major initiatives within the organization.

Of particular note, the work described here on infrastructuring the PCORnet® node, combined with an organizational commitment to its sustainability, led to establishing a network-based research unit (NBRU) within the Clinical and Translational Science Awards at Michigan. The NBRU now serves as a liaison between U-M investigators and federated research studies and maintains the informatics infrastructure and curates the data associated with four national research networks: the Trial Innovation Network, the Midwest Area Research Consortium for Health (MARCH), Accrual to Clinical Trials (ACT), and PaTH/PCORnet® Network (https://michr.umich.edu/nbru-overview).

Applying a theoretical lens to our study, we bring forward a perspective on how node infrastructure comes into existence through the process of infrastructuring, where sociotechnical relationships among people, technology, and organizations are formed and maintained. We applied an integrated perspective of infrastructuring along the dimensions of enabling technology, organizing work, and institutionalizing, and across specific considerations of aligning end goals, motivating and ensuring participation, and designing for use. This approach makes apparent factors that we have not identified in the literature on federated research and data networks in health care.

The questions we put forward provide a language and guide for planning, developing, and sustaining the infrastructure of a node as a local institution participates in a federated research network. Key insights include that infrastructuring a node on a FR&DN requires that new configurations of teams with cross-disciplinary expertise should be formed; existing resources should be leveraged to design and assemble a node; substantial effort must be dedicated to communication, collaboration, and mediation within a complex and dynamic node governance structure – including the organization, the CDRN, the FR&DN, and the technical network; and it may be particularly useful to approach infrastructure and infrastructuring a node from a sociotechnical-centric perspective, not solely a technology-centric perspective.

Limitations of the study are recognized. Our single case may not be fully generalizable to other organizations and networks. Indeed, different CDRNs have very different structures. For example, One Florida builds on an existing highly centralized data resource that encompasses data from multiple clinical agencies. However, it is not known what work was required of those clinical agencies prior to contributing data to OneFlorida. In addition, since the time of this node development, initiatives such as Smart IRBs and common Data Use Agreements have become more routine, somewhat reducing the complexity of aligning governance across organizations within a federated network. Local semantic resources that support mapping local terminology to common data models, the use of standard-based terminologies as value sets, and estimates of the impact of semantics on data quality are highly variable within and across nodes and may have impacted the nature of the work tasks that were identified and classified in this study.

Future work is needed to validate the framework and questions we propose, both at other nodes and in the context of other networks. New theoretical frameworks, concepts, and metrics need to be introduced into the literature on federated research networks in health care, reflecting capacity, and capability for infrastructuring across many different dimensions and work efforts. Cross-disciplinary collaboration is needed, and attributions of those doing the infrastructuring work are needed. The Contributor Roles Taxonomy (CrediT), now being piloted by several journals, may be helpful in this regard (https://casrai.org/credit/). Researchers studying infrastructuring for cyberinfrastructure (e.g., Computer Supported Cooperative Work (CSCW) groups), the sociology of science, team science, as well as researchers in health care should be encouraged to jointly explore infrastructuring for federated research in health care.

The work of infrastructuring nodes will continue to expand as new networks emerge and organizations participate in multiple networks, each with potentially different scales, focus, governance, technical platforms, data models, approaches to data use and sharing, funding models, and more. The work of the individual local organizations hosting nodes on research networks should not continue to be underestimated and unrecognized. The perspective of infrastructuring that we propose offers a meta-level view of activities required to design, maintain, use, and sustain node functioning on federated research networks. The questions we put forward provide a guide for planning, developing, and sustaining local institutions’ participation as a node on a federated research and data network.
